# Introducing the ‘Transforming cardiology with AI’ series

**DOI:** 10.1007/s12471-025-01948-1

**Published:** 2025-03-28

**Authors:** Pim van der Harst

**Affiliations:** Department of Cardiology, Division of Heart and Lungs, University Medical Centre Utrecht, Utrecht, The Netherlands

Artificial intelligence is becoming an integral part of todays’ cardiology, offering new possibilities for diagnosis, treatment, hospital and home monitoring, self-care, clinical decision support, and more. AI has the potential to enhance workflow efficiency, risk stratification, imaging interpretation, and personalised medicine, ultimately benefiting physicians, patients, and healthcare systems. As these technologies continue to evolve, their real-world clinical value remains a subject of discussion and ongoing investigation.

To explore cardiovascular developments, the *Netherlands Heart Journal* is launching the ‘Transforming cardiology with AI’ series. This series will focus on recently available AI-driven technologies, providing a concise evaluation of their clinical utility, performance, and potential limitations. This content is not intended to be promotional but rather reflects the authors’ personal perspectives on the practical. We also invite our readership to submit suggestions or inquiries regarding AI-driven technologies they have tested, to be featured in this series. Potential topics should involve commercially available products with regulatory approval (e.g. CE marking, UE MDR, FDA clearance, or equivalent).

The first article in this series explores an AI-driven digital stethoscope, evaluating its potential role in cardiovascular diagnostics, its integration of ECG recording, sound amplified and AI-assisted auscultation, as well as the validation challenges related to its added value in terms of outcomes and accessibility.

This issue also presents three Dutch studies on cardiovascular disease research and clinical care. The DECIPHER-PLN study investigates phospholamban (PLN) R14del cardiomyopathy, using a multi-omics approach to to identify disease pathways and biomarkers [1]. The PENELOPE study evaluates the long-term impact of a protocol-led LDL-C-lowering strategy in post-myocardial infarction patients, highlighting the challenges in lipid control [2]. Lastly, a substudy of the AIDA trial examines lesion preparation and stent optimisation in PCI with drug-eluting stents, showing that oversized predilatation balloons increase the risk of lesion-oriented events and suggesting differences between diabetic versus non-diabetic patients [3].
